# A Methodology for the Hybridization Based in Active Components: The Case of cGA and Scatter Search

**DOI:** 10.1155/2016/8289237

**Published:** 2016-06-14

**Authors:** Andrea Villagra, Enrique Alba, Guillermo Leguizamón

**Affiliations:** ^1^Universidad Nacional de la Patagonia Austral, Ruta 3 Acceso Norte, s/n, Caleta Olivia, 9011 Santa Cruz, Argentina; ^2^Universidad de Málaga, Campus Teatinos, 29071 Málaga, Spain; ^3^Universidad Nacional de San Luis, Ejército de Los Andes 950, 5700 San Luis, Argentina

## Abstract

This work presents the results of a new methodology for hybridizing metaheuristics. By first locating the active components (parts) of one algorithm and then inserting them into second one, we can build efficient and accurate optimization, search, and learning algorithms. This gives a concrete way of constructing new techniques that contrasts the spread ad hoc way of hybridizing. In this paper, the enhanced algorithm is a Cellular Genetic Algorithm (cGA) which has been successfully used in the past to find solutions to such hard optimization problems. In order to extend and corroborate the use of active components as an emerging hybridization methodology, we propose here the use of active components taken from Scatter Search (SS) to improve cGA. The results obtained over a varied set of benchmarks are highly satisfactory in efficacy and efficiency when compared with a standard cGA. Moreover, the proposed hybrid approach (i.e., cGA+SS) has shown encouraging results with regard to earlier applications of our methodology.

## 1. Introduction

Designing increasingly efficient algorithms to solve complex problems of both optimization and search is traditionally an important aspect of research in computer science. One main objective in computer science is (primarily) to develop new methods that are able to solve complex problems with a very low computational effort, thus improving upon the results obtained by existing algorithms. Interestingly, this allows current problems to be addressed more efficiently and also permits address tasks that were unsolved in the past due to their high computational cost. In this context, research into metaheuristics to solve complex optimization problems is currently expanding [1–3].

In the field of optimization there is an important interest in hybrid metaheuristics [4]. The vastly increasing number of reported applications and dedicated scientific events on hybrid metaheuristics document the popularity, success, and importance of this specific line of research. Satisfactory results have been obtained in many classical optimization and real life problems using hybrid metaheuristics. There are various characterizations of hybrid metaheuristics. Raidl in [5] shows a classification that combines the taxonomy proposed by Talbi in [6] with the point of view of [7, 8]. Classifications with respect to parallel metaheuristics are partly adopted from [9] and with respect to the hybridization of metaheuristics with exact optimization techniques from Puchinger and Raidl [10].

Talbi [6] presents two classifications for hybrid algorithms: hierarchical and flat. This classification establishes specific hybrid schemes where, in general, different algorithms are combined according to certain criteria. In contrast to the standard way of hybridization described in the above cited articles, the methodology used in this paper (firstly proposed in [11]) presents an unconventional way of hybridization. The aim of this hybrid approach is to create algorithms at which design follows similar criteria to those established for designing hybrid algorithms, but incorporating components of other algorithms rather than the algorithm as a whole. Thus, the goal of this paper is to apply the methodology proposed in [11] in new domains and algorithms. In this work we identify active components (ACs) of Scatter Search [12]. These active components represent the functional parts for SS that are vital for the appropriate performance of it. Then we enhance a cGA [13–15] with the identified active components of SS. The results obtained by the hybrid cGA improve in quality, number of evaluations, and time with respect to those obtained by the standard cGA. In addition, in this work and unlike previous work, we obtain improvements regarding the hit ratio and mainly the time required to reach the optimum.

This paper is organized as follows: Section 2 describes the algorithms cGA and Scatter Search.  Section 3 demonstrates the application of the novel methodology to identify active components of Scatter Search and the problems chosen to be resolved by the algorithms.  Section 4 discusses our hybrid algorithms that enhance cGA with the active components of Scatter Search identified by the proposed methodology. Sections 5 and 6 describe the experiments and results, and finally Section 7 draws some conclusions and suggests some future lines of research.

## 2. Characterizing Algorithms

In this section we describe the standard algorithms used in this paper. Firstly we present the host metaheuristic cGA. Secondly, we describe the SS metaheuristic that we chose to enhance cGA.

### 2.1. Celullar Genetic Algorithm

A cGA [13] is a subclass of Genetic Algorithms (GAs) in which the population is structured in a specified topology, so that individuals may only interact with their neighbors. These overlapping small neighborhoods help in exploring the search space because the induced slow diffusion of solutions through the population provides a kind of exploration, while exploitation takes place inside each neighborhood via genetic operations. In cGAs the population is usually structured in a 2D toroidal grid. The most commonly used neighborhood structure is L5 [15].

In Algorithm 1 we present the pseudocode of a standard cGA. It starts by generating and evaluating an initial population. Then, genetic operators (selection, recombination, mutation, and replacement) are iteratively applied to each individual until the termination condition is met.

The population is structured in a two-dimensional (2D) toroidal grid (gridcGA), and the neighborhood defined on it contains five individuals (line (4)). The individual being considered (individual (*x*, *y*)) is always selected to be one of the two parents (line (5)). The second parent is selected by Tournament Selection (line (6)). Genetic operators are applied to individuals in lines (7) and (8). Here we use a two-point crossover operator (DPX1) which produces one single individual (the one with the largest part from its best parent) and the traditional binary mutation operator (bit-flip).

After that, the algorithm computes the fitness value of the new offspring individual (line (9)) and inserts it, replacing the current individual in the population (line (10)) following a given replacement policy.

### 2.2. Scatter Search Algorithm

Scatter Search (SS) is a population-based metaheuristic first introduced by Glover [12]. SS has been shown to be efficient in solving a wide range of combinatorial and nonlinear optimization problems [16, 17]. It operates on a set of solutions, called the reference set, which constitutes good solutions obtained from the previous search. The aim of SS is to derive new solutions from combined solutions in order to yield better solutions. The basic components of SS are as follows: (a) there are a set of solutions with a certain degree of quality and diversity (called *P*). From this set the reference set (called *R*) is extracted (generally |*P* | ≥10 × |*R*|); (b) a reference set is extracted from *P* with the criterion of “forming a set of different quality solutions.” These solutions are different in quality and diversity; (c) a combination method that combines all solutions in the reference set (here we apply DPX1); and (d) an improved method like a local search enhances not only the combined solutions but also the reference set (here we use Hill-Climbing).

In Algorithm 2 we present the pseudocode of a standard SS. It starts by generating and evaluating an initial population *P* (line (1)). Then the solutions in *P* are enhanced with a local search method (line (2)) and these enhanced solutions (*P*′) are used to create the reference set *R* of size *b* (line (3)). The reference set *R* is formed by two subsets named *R*1 and *R*2. The subset *R*1 contains *b*/2 best solutions of *P*′ and *R*2 contains *b*/2 the most diversity solutions with respect to the solutions in *R*1. Then, until the criterion of termination is reached (line (4)) the following lines are performed (lines (5)–(7)): the solutions in *R* are recombined. The recombined solutions are enhanced and these enhanced solutions are used to update the reference set. The enhanced solutions replace the solutions in the subset *R*1 if they are better in quality than the solutions currently in the subset *R*1. The enhanced solutions replace the solutions in the subset *R*2 if they are better in diversity than the solutions currently in subset *R*2.

Something to take into account is that if no new recombined solutions update the subset *R*1 and the termination condition is not reached, then subset *R*2 can be regenerated in the following way: a new population of diversity solutions is created and all solutions in subset *R*2 are replaced.

**Figure 1:**
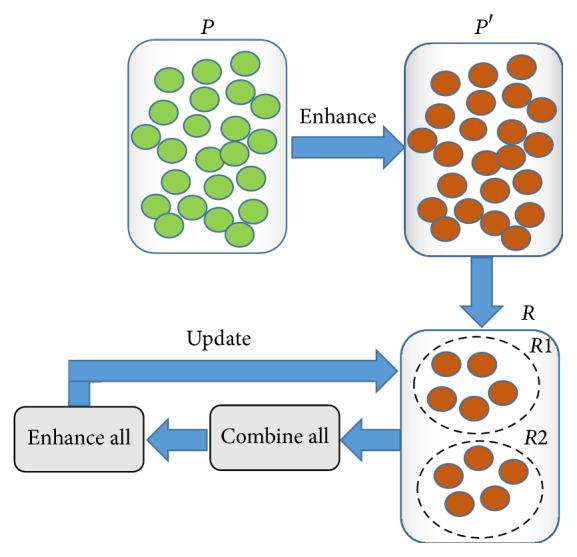
Basic scheme of Scatter Search.

Figure 1 shows a basic scheme of Scatter Search with the principal components involved in this metaheuristic: The set of initial solutions (*P*), the set of enhanced solutions (*P*′), the reference set (*R*), and the processes of combining, enhancing, and updating *R*.

## 3. Identifying Active Components of Scatter Search

In this section we identify the active components of Scatter Search, by applying the methodology proposed in [11]. Before this, we describe the problems chosen and used in the experiments presented at the end of this paper.

### 3.1. Description of the Problems

We present the set of problems chosen for the application of the proposed methodology and for the study of our cGA algorithm enhanced by active components of SS. We have decided to deal with a representative set of problems to better study our proposal (enlarged with respect to previous studies for a better assessment). The benchmark contains many different interesting features in optimization, such as epistasis, multimodality, and deceptiveness. The problems used are the Massively Multimodal Deceptive Problem (MMDP) [18], Frequency Modulation Sounds (FMS) [19], Multimodal Problem Generator (P-PEAKS) [20], COUNTSAT [21] (an instance of MAXSAT [22]), Error Correcting Code Design (ECC) [23], Maximum Cut of a Graph (MAXCUT) [24], the Minimum Tardy Task Problem (MTTP) [25], the OneMax Problem (ONEMAX) [26] (or BitCounting), and, finally, the Subset Sum Problem (SUBSETSUM) [27].

MMDP has been specifically designed to be difficult to deal with by evolutionary algorithms; FMS is a real problem in engineering P-PEAKS a generator of problems that eliminates the possibility of hand tuning algorithms for a particular problem, thereby permitting greater justice to compare algorithms. ONEMAX problem is used as a baseline for comparison. ECC is a problem of real interest in secure communications; MAXCUT, COUNTSAT, MTTP, and Subset Sum are problems of interest in operational research. In all of these problems we are maximizing the objective function, except for FMS and Subset Sum where we are minimizing. All of these problems share as a distinguishing feature their difficulty and potential real application. However, not all of them are used in complex instances: a few some simpler instances are studied to include different complexity levels that will allow us to better understand scalability and differentiate behavior. We use one-instance problem for ECC, P-PEAKS, COUNTSAT, and SUBSETSUM so we named the respective instance problems as ecc, p-peaks, countsat, and ss1000. For ecc we will consider a triple (*n*, *M*, *d*), where *n* is the length of each codeword (number of bits), *M* is the number of codewords, and *d* is the minimum Hamming distance between any pair of codewords. We consider in the present paper for this instance *M* = 24 and *n* = 12. In the case of p-peaks as the idea is to generate *P* random *N*-bit string that represents the location of *P* peaks in the search space we use here *P* = 100 peaks of length *N* = 100 bits each. Regarding countsat the solution value is the number of clauses that are satisfied by *n*-bit input string. In this work, we use for this instance *n* = 20. Finally, for ss1000 and taking into account the characteristics of this problem we define an instance size of 1000 integers. FMS consists of six real-parameters of frequency modulated sound model with parameters defined in the range [−6.4, +6.35]; we encode each parameter into a 32-bit substring in the individual. We use two instances fms32x6 and fms48x6 (with bit strings of 32 and 48 bits, resp.). MMDP is composed of *k* deceptive subproblems; we use two instances mmdp40 and mmdp60 (with *k* = 40 and *k* = 60 subproblems, resp.). MAXCUT problem consists in dividing a weighted graph into two disjoint subgraphs so that the sum of the weights of the shafts having one end of each subset is maximized. To encode a partition of vertices we use a binary string where each digit corresponds to a vertex. We have defined three instances named cut20.01, cut20.09, and cut100 where the first one is a sparse graph with 20 vertices, the second one is a dense graph with 20 vertices, and the third one is a scalable graph with 100 vertices. For MTTP three instances were considered and named mttp20, mttp100, and mttp200 (with size of 20, 100, and 200, resp.). Finally, for ONEMAX we use two instances named onemax500 and onemax1000 (with size of 500 and 1000, resp.).

In this paper we use the aforementioned set of problems with a binary representation, but in the near future we expect to extend the set of problems to other representations (e.g., permutations) and also to another set of continuous problems.

### 3.2. Active Components in SS

*Observation* is the first step to take into consideration in any research study, as the* scientific method* suggests. Obviously it is the first activity we need to undertake to clarify how the metaheuristic works and to try to detect its strengths and weaknesses.

After studying the metaheuristic, we have used common sense and our knowledge of the behavior of SS to identify candidate active components: we propose as candidate active components subsets *R*1 and *R*2. That is to say, set *R* = {*R*1, *R*2} is the candidate active component of SS.

Then we need to apply the two mechanisms proposed in the methodology to determine whether or not the candidate part of the algorithm is really an active component that directly affects the behavior of SS. These two mechanisms are first to numerically check the contribution of candidate active components and then to make software profiling to complement/validate the numerical results.

To analyze the behavior of SS before and after removing parts of it, we define the following variants:

(a) SS: the first algorithm is the complete SS, that is, a regular SS.
(b) SS-WEP: the set *P* without enhancing its solutions, that is, a population *P*, is created without improving its solutions.
(c) SS-OR2: create the set *R* only with the subset *R*2; that is, *R* is created without *R*1.
(d) SS-OR1: create the set *R* only with the subset *R*1; that is, *R* is created without *R*2.
(e) SS-WER: the new solutions generated after recombining each solution in *R* are not enhanced. That is to say, its new solution, produced by the recombination of every other solution in *R*, is not improved.
(f) SS-WRR: none of the solutions in set *R* are recombined or improved.

For each case described above, we performed 150 independent runs.  Table 1 shows the average percentage of success in each case.

In Table 1 we can see that the first three hybrids obtained on average the best percentage of success. The last three variants obtain on average the lowest percentage of success: this means that the removed parts are really important for the algorithm. Hence, the subset *R*1, the subset *R*2 (i.e., set *R*), the recombination, and improvement of the solutions after recombination are extremely important for the good behavior of SS. In summary, the set *R*, the process of recombination of the solutions in *R*, and the improvement of the new solutions are active components of SS.

After detecting which are the active components from a numerical point of view, we now proceed to confirm from a software point of view the previously detected active components. To do so, we then apply a profiling mechanism. We decided to pay particular attention to the time in ms consumed by each candidate active component detected. We performed 150 independent runs of SS for mmdp40.  Table 2 shows the average and median values for the percentage of time consumed in seconds.

The first column represents the process of generating the initial population (called* GenerateP*); the second column represents the process of enhancing the initial population (called* EnhanceP*). The third column depicts the generation of set *R*; the fourth column depicts the results from recombining set *R*. The fifth column demonstrates the improvement of process the new solutions after the recombination. The sixth column stands for the updating of set *R* with the new solutions generated. Finally, the last column represents other computational operations that are not involved in the candidates' active components.

We can observe that the process of enhancing the solutions after the recombination (*EnhanceR*) is the most time consuming process. Nevertheless, the other processes (*GenerateR*,* RecombinateR*, and* UpdateR*) are not time consuming. Our knowledge of SS and common sense tells us that these processes (*GenerateR*,* RecombinateR*, and* UpdateR*) are necessary for the process that consume most of the time.

After applying the methodology and from the results obtained we can claim that in SS the following active components have been identified: set *R* with the processes of (a) recombination, (b) improvement, and (c) actualization (*UpdateR*). In the following section we show how we can apply the detected active components of SS to create a better algorithm, in this case an improved cellular GA.

## 4. Hybrid cGA with Active Components of SS

After applying the methodology we found that set *R*, in combination with the processes of recombination, improvement, and actualization, is the active components of SS. Then we proceed to incorporate these components inside a cGA in order to build a new hybrid algorithm hopefully better than its components. To do so we can plug the set *R* into a base cGA in the following way. We know that *R* is formed by two subsets *R*1 and *R*2, so in our hybrid (called hySSA-rec) *R*1 is created with the neighbors of the target individual and *R*2 is created with random individuals (in each generation).

In Algorithm 3 we present the pseudocode of our hybrid algorithm hySSA-rec. It starts by generating and evaluating an initial population (the standard cGA). Then (for each individual) subset *R*1 is generated containing the neighbors of the target individual (individ (*x*, *y*)) (line (4)). Subset *R*2 is created with random individuals (line (5)). Then *R*1 and *R*2 conform to set* R* (line (6)). Afterwards, the target individual recombines with each individual in* R* (line (7)). The recombined method used is DPX1. Then each new solution generated by the recombination is evaluated (line (8)) and improved (line (9)) and the best solution is selected (line (10)). Finally, the algorithm inserts the best solution (*bestSol*) replacing the current individual in the population (line (11)) following a given replacement policy. In this paper the criterion of replacement is “if no worse than.”

**Figure 2:**
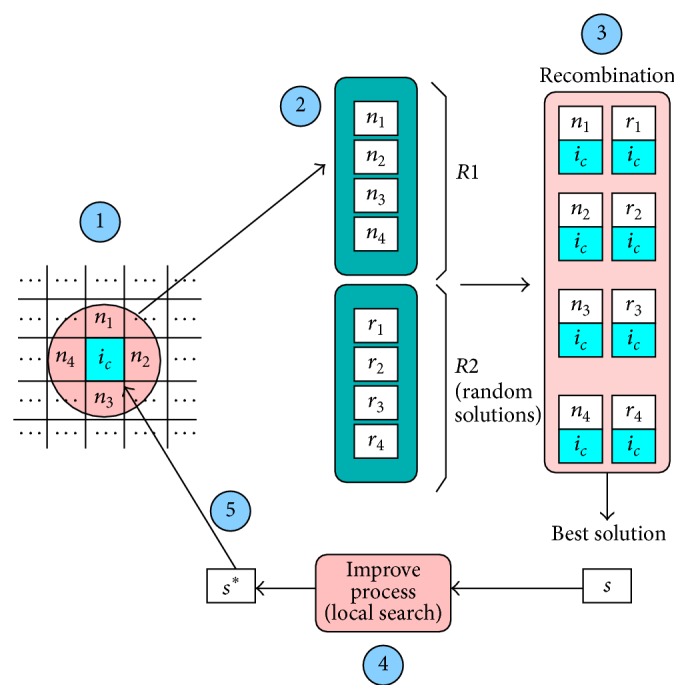
Active component of SS into cGA (hySSA-rec).

Figure 2 shows the flow of the proposed algorithm hySSA-rec. We can see the following steps: in step ① the target individual and the respective neighbors (circle) are identified. Then in step ②, set *R* is defined as containing sets *R*1 (neighbors of the target) and *R*2 (random solutions). In step ③ the recombination process takes place where the target individual recombines with each solution in *R*. After that (step ④) the best solution is enhanced with a local search process. Finally, in step ⑤ the target individual is replaced by the new solution if the replacement policy is satisfied (i.e., if the new solution is not worse than the previous one).

## 5. Experiments and Analysis of Results

In this section we present the experiments and the analysis of results obtained by the proposed algorithm. Firstly we describe the parameterization used in both algorithms. Secondly, we present the experiments carried out to analyze the proposed algorithm. Our goal is to show that the used methodology that takes into account active components of a given metaheuristic can create in a structured manner efficient and accurate algorithms.

### 5.1. Parameterization

The parameterization used for the standard cGA is the following one: (a) 400 individuals for population size; (b) one of the parents that is the current individual being considered in the loop of cGA, while the other one is obtained by using Tournament Selection, (c) DPX1 (which, starting from two parents, obtains one single new individual with the longest portion of the best of the two parents) for recombination with a probability set to 1.0, and (d) bit-flip mutation for the standard cGA with a probability of mutation set to 1/(length of chromosome). The exceptions are countsat, where we use *p*_*m*_ = (*L* − 1)/*L* and the fms32x6 and fms48x6 instances, for which a value of *p*_*m*_ = 1/(2*∗L*) is used. These two values are needed because the algorithms had a negligible solution rate with the standard *p*_*m*_ = 1/*L* probability in our preliminary set of experiments, and (e) the criterion of replacement that is “if no worse than.” For our hybrid algorithm we maintain the population size (400) individuals as well as the replacement criterion, but, given the characteristics of SS, the mutation operation is replaced by the random individuals generated in set *R*2 (this set already incorporates a kind of mutation) and the selection operator used is to select the best solution.

We start reporting results by measuring the hit rate. The cost of solving an instance problem is analyzed by measuring the number of evaluations of the objective function made during the search (only accounting for the runs having success in finding the optimum). The stop condition for all algorithms is to find a solution or to achieve a maximum of one million function evaluations. Throughout the paper all best values are marked in bold.

In all the experiments we have analyzed the conditions that must be met to use parametric tests and nonparametric tests for the statistical analysis with a confidence of *p* = 0.05 using SPSS (http://www.spss.com/). Statistically significant differences between the algorithms are shown with the symbol “•.”

All the algorithms are implemented in Java and run on a 2.53 GHz Intel i5 processor with Windows 7. We performed 150 independent runs for all algorithms and instance problems.

### 5.2. Experiments

First of all we wanted to corroborate that the instance problems we selected were indeed complex and also that the algorithms could adequately manage the instance problems. To do this we implemented Random Search (RS), Hill-Climbing (HC), and Iterated Local Search (ILS) algorithms. We show the results obtained by them as a first experiment to better know our hybrid proposal in relation to algorithms commonly found in literature.

In Table 3 we include the percentage of success in 150 independent runs for RS, HC, and ILS. We can observe a low average of success rate (20%) for RS while ILS gets the higher average of success rate, with HC lying in the middle in terms of hit percentage. Only in four instances (countsat, cut20.01, cut20.09, and mttp20) does the Random Search algorithm obtain a value of success different from 0. We of course know that RS is not a competitive algorithm (should not be at least) but we do report results with it as a sanity check for our proposal and also because it is important in science to back intuitions with concrete numerical evidence, as we do here. Also, the HC algorithm (which is expected to be more competitive than RS) obtained a low average of success compared with the ILS algorithm.

In second experiment, we will focus on comparing the standard cGA with the hybrid hySSA-rec. In Table 4 we show the percentage of success in 150 independent runs. We can see that the success rate for the proposed hybrid algorithm is higher than cGA (82% success rate obtained by hySSA-rec versus 70% success rate obtained by cGA). We can observe that hySSA-rec obtains 100% success rate in 12 problem instances out of 16 used.

As a third experiment, in Table 5 we analyze the selected performance variables, number of evaluations (*Evals*) and time (in ms). To do that we grouped the algorithms that obtained a success rate of more than 20% (that also matches a sample size of 30 successful runs for our further statistical analysis).

The first column (*instance*) represents the name of the problem resolved and the second column (*best*) has the best solution found, and then for each algorithm (hySSA-rec and cGA) the number of evaluations (columns* Evals*) needed to solve each problem and the time consumed (in ms, columns* time*). Finally, the last column (T|UMW) represents the *p* values computed by performing a* t*-test or a Mann Whitney *U* test (normal versus nonnormal distributions) as appropriate, on the time and evaluation results, in order to assess their statistical significance (columns* Evals* and* time*). We consider a 0.05 level of significance. Statistical significant differences between the algorithms are shown with the symbol “•,” while “–” marks the results with no warranties of being different from each other.

As a first main conclusion, we can observe that the number of evaluations needed to reach the optimum value is smaller for our hybrid hySSA-rec in nine out of ten problems and furthermore there are statistically significant differences between the results obtained by hySSA-rec for eight out of ten problems (ecc, p-peaks, mmdp40, cut20.01, cut20.09, mttp20, onemax500, and onemax1000). Taking into account the performance variable* time* we can observe that the behavior of our hybrid algorithm is also better than cGA in nine out of the ten problems. This is very remarkable, since most hybrids in the literature do better numerically speaking but not in real time. Additionally, the difference is statistically significant between the proposed hybrid algorithm and the cGA for eight (ecc, mmdp40, ss1000, cut20.01, cut20.09, mttp20, onemax500, and onemax1000) out of ten problems.

In an additional analysis we apply the Wilcoxon signed rank test for the number of evaluations and time.  Table 6 shows (resp.) the results obtained for the *R* ^+^, *R* ^−^, and *p* value values when comparing the two algorithms.

In Table 6 we can see that there exist statistical differences between hySSA-rec and cGA for the number of evaluations and for the efficiency (time), so we can state that also the difference is statistically significant between hySSA-rec and cGA.

**Figure 3:**
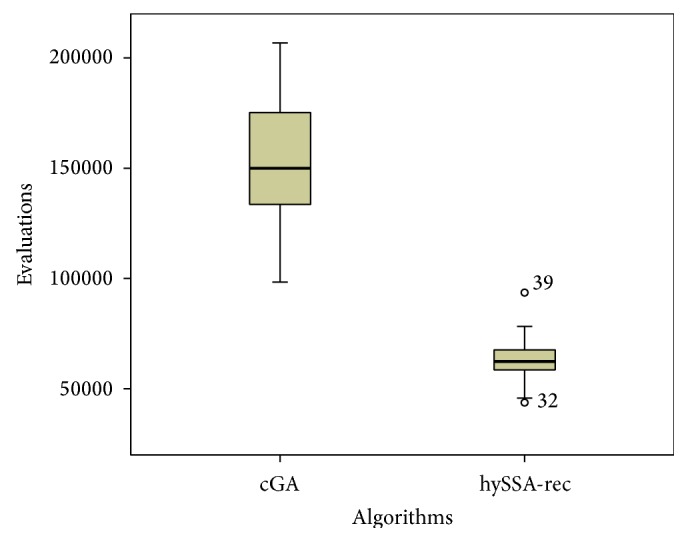
Box-plots of the number of evaluations required for the cGA and hySSA-rec to solve the ecc instance.

**Figure 4:**
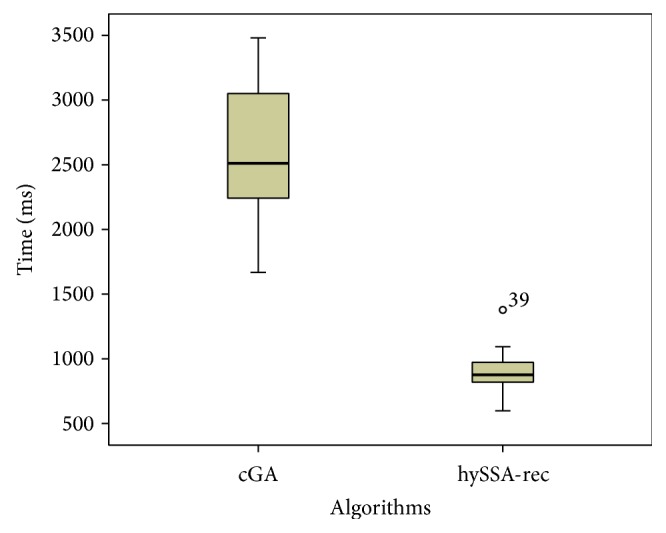
Box-plots of the time required for the cGA and hySSA-rec to solve the ecc instance.

From another perspective, Figures 3 and 4 show, respectively, the number of evaluations and the time needed for each algorithm to reach the optimum value for ecc instance. Since we have many problems and algorithms we just have selected this result as a representative case of what usually happens also for the rest of problems. We show the median values and the distributions of the results (box-plot) for each algorithm. In both figures we can see the differences obtained by our proposed hybrid algorithm against the standard cGA. The proposed hybrid algorithm reaches the lowest median values, which is a very competitive result.

## 6. Additional Analysis

In this subsection we present an additional comparison of hySSA-rec with several state-of-the-art techniques for the same instances found in [11]. In Table 7 we show the percentage of successes obtained by

- hyAS-Sel: a hybrid cGA that uses active components of Simulated Annealing (SA) as the mechanism of selection,
- hySSA-rec: the hybrid cGA proposed in this paper,
- hyAPM-local: a hybrid cGA with active components of a local Particle Swarm Optimization (PSO) as a mutation operator,
- hyAS-APM-local: a hybrid cGA with active components of SA applied to the selection operator and active components of PSO used as a mutation operator,
- cGA: the standard cGA.

We can see that the hybrids hyAS-APM and hySSA-rec are the algorithms with the best percentage of success on average (they are dead heat). Algorithms hyAS-Sel and hyAPM-local lay in the middle with the best percentage of success while the lowest one was obtained by cGA.

Now we compare the number of evaluations required by each algorithm (i.e., their numerical performance). We select the problems where all algorithms obtain a percentage of success different from 0%.  Table 8 shows the number of evaluations required for each algorithm. We can observe that our proposal in the present paper, hySSA-rec, obtains the minimum number of evaluations in eight out of ten problems.

In an additional analysis we apply a Friedman test (see [28]). This test ranks algorithms for each problem separately and it is the analog of the repeated measures ANOVA in nonparametric statistical procedures; therefore, it is a multiple comparisons test that aims to detect significant differences between the behavior of two or more algorithms. The null hypothesis for the Friedman test states equality of medians between the populations. The alternative hypothesis is defined as the negation of the null hypothesis, so it is nondirectional. This test ranks the algorithms for each problem separately; the best performing algorithm should have the rank of 1, the second best one rank of 2, and so on. The null hypothesis states that all the algorithms behave similarly (i.e., their respective ranks should be equal).  Table 9 shows the results of the Friedman statistic (distributed according to chi-square with 4 degrees of freedom: 18). The *p* value calculated by the Friedman test is 0.001 (much lower than 0.05), and this means that there are statistically significant differences between the results obtained by algorithms. We can also observe that the best rank (the smallest value) is obtained by hySSA-rec (an expected result because we observe that hySSA-rec always requires the minimum number of evaluations). The worse rank (the highest value) is obtained by hyAS-APM (also an expected result because this hybridization requires more processing time than the other hybrids). As a summary, we honestly report our proposal as the best. The strength of this work is not only to provide accurate and efficient hybrid algorithms, but it is still rather more important to give a successful methodology to guide other researchers in a way of building them.

**Figure 5:**
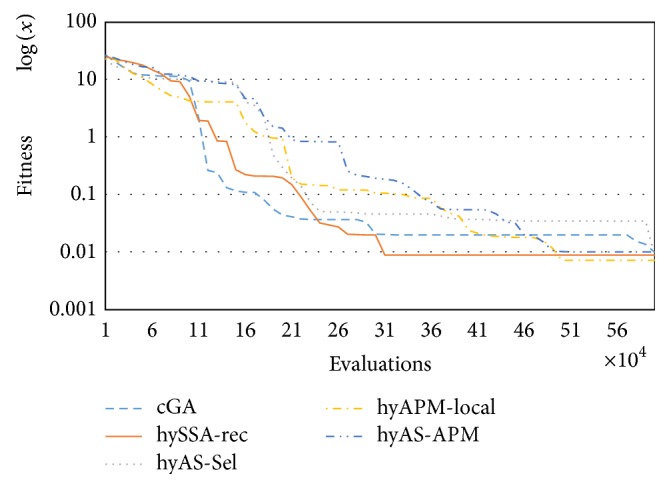
Convergence for all the algorithms for a representative case (fms32x6).

From another perspective, we choose a representative instance just to illustrate how our proposed hybrid converges faster to the optimum, both in number of evaluations and in real time, than the standard cGA. In Figure 5 we can observe this behavior. Also hyAPM-local and hyAS-APM converge faster than cGA.

In summary, our results support our contributions that are numerous and varied: (i) we have a working methodology to build new hybrid algorithms, (ii) this methodology yields fast algorithm with respect to other hybrids and the basic merged algorithms, (iii) the resulting techniques are competitive with respect to the state of the art, and (iv) we have tested the base hypothesis of this new hybridization in problems appearing in engineering, operations research, and communications and tested their scalability with respect to number of variables and constraints.

## 7. Conclusions and Future Work

The motivation for this work was to improve the performance of a basic cGA with the addition of components of Scatter Search, with the goal of creating a hybrid algorithm that would improve upon the results already obtained by the core technique.

We have also applied the methodology we proposed in a previous article to identify active components of a metaheuristic. We have then provided a competitive algorithm by following a methodology to do so: an additional contribution. This methodology offers a scientifically structured alternative of building future algorithms in a field (hybridization) where wild combinations of algorithms are the usual way to go.

From the results obtained in the experiment we can claim that our hybrid algorithm obtains higher success percentages (number of runs locating the optimum of the problem) than those obtained by cGA in most of the instances analyzed. Moreover, in most instances the best performance in terms of the number of evaluations and also in time was obtained by the mentioned hybrid algorithm. This means that our hybridization framework can effectively improve the efficiency of a basic algorithm by detecting and embedding active components from other techniques inside it. We can also state that the new algorithm is often better than the standard one and even competitive with existing good results for the same problems.

These results encourage us to expand the set of problems discussed in future approaches, to find other active components from other metaheuristics, and to improve and extend the methodology to identify active components.

## Figures and Tables

**Algorithm 1:**
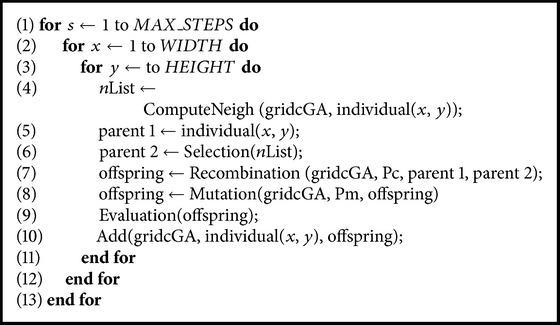
Pseudocode of a cGA.

**Algorithm 2:**
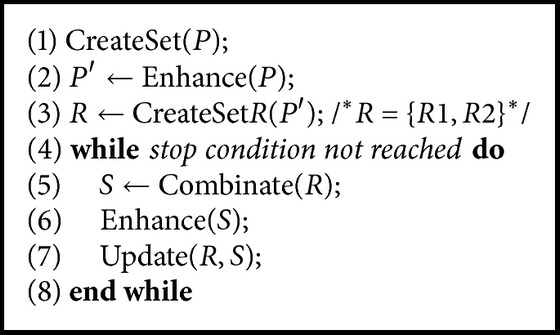
Pseudocode of a SS.

**Algorithm 3:**
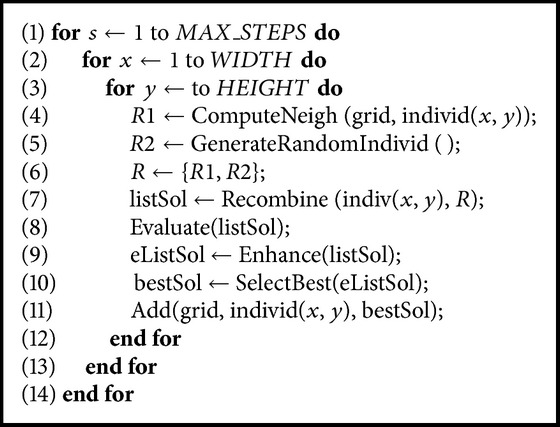
Pseudocode of a hySSA-rec.

**Table 1:** Percentage of success obtained by SS, SS-WEP, SS-OR2, SS-OR1, SS-WER, and SS-WRR.

Instance	SS	SS-WEP	SS-OR2	SS-OR1	SS-WER	SS-WRR
e cc	**55%**	**75%**	45%	0%	25%	37%
mttp100	**100%**	99%	**100%**	0%	0%	0%
onemax500	**100%**	**100%**	**100%**	0%	0%	**100%**

Average	**85%**	**91%**	**82%**	0%	8%	46%

**Table 2:** Profiling analysis in percentage of time consumed (in seconds) of SS for m mdp40.

	Generate*P*	Enhance*P*	Generate*R*	Recombinate*R*	Enhance*R*	Update*R*	Other activities
Average	0.96%	18.82%	0.78%	0.92%	**63.65%**	0.63%	14.24%
Median	0.73%	17.25%	0.58%	0.60%	**72.54%**	0.49%	10.43%

**Table 3:** Percentage of successes obtained by Random Search, Hill-Climbing, and Iterated Local Search Algorithms.

Instance	RS	HC	ILS
e cc	0%	47%	**48%**
p-peaks	0%	**100%**	**100%**
countsat	**57%**	2%	5%
ss1000	0%	**100%**	**100%**
fms32x6	0%	0%	0%
fms48x6	0%	0%	0%
mmdp40	0%	0%	0%
mmdp60	0%	0%	0%
cut20.01	**100%**	49%	52%
cut20.09	**100%**	47%	51%
cut100	0%	79%	**85%**
mttp20	**63%**	**100%**	**100%**
mttp100	0%	**97%**	**97%**
mttp200	0%	5%	**11%**
onemax500	0%	**100%**	**100%**
onemax1000	0%	97%	**100%**

Average	20%	51%	**53%**

**Table 4:** Percentage of success obtained by hySSA-rec and cGA.

Instance	hySSA-rec	cGA
e cc	**100%**	**100%**
p-peaks	**100%**	**100%**
countsat	**100%**	0%
ss1000	**100%**	**100%**
fms32x6	**53%**	25%
fms48x6	**42%**	11%
mmdp40	**100%**	42%
mmdp60	**100%**	1%
cut20.01	**100%**	**100%**
cut20.09	**100%**	**100%**
cut100	**100%**	47%
mttp20	**100%**	**100%**
mttp100	14%	**100%**
mttp200	0%	**100%**
onemax500	**100%**	**100%**
onemax1000	**100%**	**100%**

Average	**82%**	70%

**Table 5:** Results obtained by our hybrid hySSA-rec and cGA algorithms for a set of problems.

		hySSA-rec		cGA		T∣UMW	
Instance	Best	Evals	Time	Evals	Time	Evals	Time
e cc	0.07	**62379**	**876**	150000	2512	•	•
p-peaks	1.00	**26711**	3447	39200	**3283**	•	—
mmdp40	40.00	**13903**	**75**	143200	2295	•	•
ss1000	0.00	102747	**2006**	**102000**	7128	—	•
cut20.01	10.12	**3910**	**8**	4800	17	•	•
cut20.09	56.74	**4943**	**11**	8000	49	•	•
cut100	1077.00	**184375**	**3108**	193600	3734	—	—
mttp20	0.02	**3620**	**5**	5600	28	•	•
onemax500	500.00	**20797**	**217**	128200	3581	•	•
onemax1000	1000.00	**33100**	**793**	247400	15998	•	•

**Table 6:** Wilcoxon signed rank test to hySSA-rec and cGA for number of evaluations and time.

	*R* ^+^	*R* ^−^	*p* value
Evals	8	1	0.013
Time	9	1	0.017

**Table 7:** Percentage of success obtained by hyAS-Sel, hySSA-rec, hyAPM-local, hyAS-APM, and cGA.

Instance	hyAS-Sel	hySSA-rec	hyAPM-local	hyAS-APM	cGA
e cc	**100% **	**100% **	**100%**	**100%**	**100%**
p-peaks	**100%**	**100% **	**100%**	**100%**	**100%**
countsat	7%	**100% **	80%	**99%**	0%
ss1000	**100%**	**100% **	**100%**	**100%**	**100%**
fms32x6	46%	53%	83%	**86%**	25%
fms48x6	34%	42%	40%	**84%**	11%
mmdp40	55%	**100% **	58%	**65%**	42%
mmdp60	0%	**100%**	**16%**	10%	1%
cut20.01	**100%**	**100% **	**100%**	**100%**	**100%**
cut20.09	**100%**	**100% **	**100%**	**100%**	**100%**
cut100	**72**%	**100% **	38%	64%	47%
mttp20	**100%**	**100% **	**100%**	**100%**	**100%**
mttp100	**100%**	14%	**100%**	**100%**	**100%**
mttp200	**100%**	0%	**100%**	**100%**	**100%**
onemax500	**100%**	**100%**	**100%**	**100%**	**100%**
onemax1000	**100%**	**100%**	0%	0%	**100%**

Average	76%	**82%**	76%	**82%**	70%

**Table 8:** Numerical performance of our hySSA-rec versus hyAS-Sel, hyAPM-local, hyAS-APM, and cGA.

		hyAS-Sel	hySSA-rec	hyAPM-local	hyAS-APM	cGA
Instance	Best fitness	Evals	Evals	Evals	Evals	Evals
e cc	0.07	160200	**62379**	141400	168400	150000
p-peaks	1.00	40000	**26711**	39600	38600	39200
mmdp40	40.00	168000	**13903**	182000	203800	155600
ss1000	0.00	234000	102747	162400	358800	**102000**
fms32x6	0.00	525800	**368668**	462800	429000	477800
cut20.01	10.12	4000	**3910**	4800	4600	5200
cut20.09	56.74	7200	**4943**	7600	8600	8000
cut100	1077.00	337800	184375	210800	344200	**180800**
mttp20	0.02	5200	**3620**	4800	4400	5600
onemax500	500.00	130000	**20797**	199200	224400	128200

**Table 9:** Average rankings of the algorithms (Friedman) taking into account the number of evaluations.

Algorithm	Ranking
(1) *hySSA-rec*	*1.20*
(2) cGA	3.00
(3) hyAPM-local	3.29
(4) hyAS-Sel	3.60
(5) hyAS-APM	4.90
